# Comprehensive Network Analysis Reveals the Targets and Potential Multitarget Drugs of Type 2 Diabetes Mellitus

**DOI:** 10.1155/2022/8255550

**Published:** 2022-07-28

**Authors:** Wan Zhou, Qiang Liu, Wei Wang, Xiao-Jing Yuan, Chun-Chun Xiao, Shan-Dong Ye

**Affiliations:** ^1^Laboratory for Diabetes, Department of Endocrinology, The First Affiliated Hospital of USTC, Division of Life Sciences and Medicine, University of Science and Technology of China, Hefei 230001, China; ^2^Institute on Aging and Brain Disorders, The First Affiliated Hospital of USTC, Division of Life Sciences and Medicine, Hefei National Laboratory for Physical Sciences at the Microscale, University of Science and Technology of China, Hefei 230026, China; ^3^The First Affiliated Hospital of USTC, Division of Life Sciences and Medicine, University of Science and Technology of China, Hefei 230026, China

## Abstract

Type 2 diabetes mellitus (T2DM) is a metabolic disease with increasing prevalence and mortality year by year. The purpose of this study was to explore new therapeutic targets and candidate drugs for multitargets by single-cell RNA expression profile analysis, network pharmacology, and molecular docking. Single-cell RNA expression profiling of islet *β* cell samples between T2DM patients and nondiabetic controls was conducted to identify important subpopulations and the marker genes. The potential therapeutic targets of T2DM were identified by the overlap analysis of insulin-related genes and diabetes-related genes, the construction of protein-protein interaction network, and the molecular complex detection (MCODE) algorithm. The network distance method was employed to determine the potential drugs of the target. Molecular docking and molecular dynamic simulations were carried out using AutoDock Vina and Gromacs2019, respectively. Eleven cell clusters were identified by single-cell RNA sequencing (scRNA-seq) data, and three of them (C2, C8, and C10) showed significant differences between T2DM samples and normal samples. Eight genes from differential cell clusters were found from differential cell clusters to be associated with insulin activity and T2DM. The MCODE algorithm built six key subnetworks, with five of them correlating with inflammatory pathways and immune cell infiltration. Importantly, CCR5 was a gene within the key subnetworks and was differentially expressed between normal samples and T2DM samples, with the highest area under the ROC curve (AUC) of 82.5% for the diagnosis model. A total of 49 CCR5-related genes were screened, and DB05494 was identified as the most potential drug with the shortest distance to CCR5-related genes. Molecular docking illustrated that DB05494 stably bound with CCR5 (-8.0 kcal/mol) through multiple hydrogen bonds (LYS26, TYR37, TYR89, CYS178, and GLN280) and hydrophobic bonds (TRP86, PHE112, ILE198, TRP248, and TYR251). This study identified CCR5 as a potential therapeutic target and screened DB05494 as a potential drug for T2DM treatment.

## 1. Introduction

Diabetes is a metabolic syndrome that affects more than 536 million people worldwide. The prevalence of diabetes is consistently growing and is expected to rise to 780 million by 2045 [1]. As a result, the high incidence and mortality dramatically increased, placing a huge burden on the health care system [2]. The main categories of diabetes include type 1 diabetes (T1DM), type 2 diabetes mellitus (T2DM), gestational diabetes, and other less common types, such as monogenic diabetes syndrome, pancreatic exocrine diseases, and diabetes caused by drugs or chemicals [3]. Among them, T2DM is the most common type of diabetes, accounting for 90% of all diabetic patients [4]. T2DM is characterized by hyperglycemia and is caused by insulin resistance, reduced or insufficient insulin secretion, and/or improper secretion of glucagon hormones [5]. Diabetes that is not effectively controlled for a long time usually causes various serious complications such as acute exacerbation of lethal diabetes pyruvate and hyperosmolar coma, in addition to severe chronic and organic lesions, such as cardiovascular disease, diabetic neuropathy, chronic kidney disease, and retinopathy. Metformin is the first-line drug for the treatment of T2DM by inhibiting liver gluconeogenesis and increasing peripheral insulin sensitivity. Additionally, sulfonylureas, thiazolidinediones, glucose-like peptide-1 (GLP-1) analogs, and dipeptidyl peptidase-4 (DPP4) inhibitors are widely used in clinical therapy [6]. However, these hypoglycemic drugs have some limitations such as long treatment time, large side effects, and high price, and there is a great demand for effective, low-toxic or nontoxic, and reasonably priced drugs for treating diabetes. Therefore, early screening and the development of efficient, safe, and sustainable drugs are still the main objectives of T2DM management [7]. For this purpose, a precise understanding of the molecular mechanism of T2DM and the key genes that play critical roles in T2DM development is a basis for further exploiting potential therapeutic drugs for T2DM.

Recent technologies and methodologies such as single-cell RNA sequencing (scRNA-seq) and molecular docking create the possibility for efficiently exploring potential drug targets. The dominant paradigm in drug discovery is the concept of designing maximally selective ligands to act on individual drug targets [8]. The emergence of functional genomics, proteomics, chemical informatics, and other system-based scientific methods has raised expectations for drug discovery and development of new targets [9]. In recent years, web-based drug discovery has been considered a promising method for cost-effective drug development [10]. Network pharmacology analyses consider drug responses in the context of cellular or phenotypic networks [11]. Some studies have developed drugs with certain therapeutic effects on specific diseases through the application of network pharmacology, but these studies are not comprehensive enough as there was a lack of taking the influence of T2DM heterogeneity into consideration [12].

Molecular docking is a widely used technique for large-scale virtual screening of the interactions between small-molecule ligands and their target proteins [13]. Docking can identify new compounds with therapeutic significance, predict ligand-target interactions at the molecular level, or depict the structure-activity relationship, without knowing the chemical structure of other target regulators in advance [14]. Recently, in silico approaches which compute the dynamic motion of atoms and molecules with respect to time were widely used and had made some progress in the screening of the target of finding drugs for diseases and the mechanism exploration for medicine [15]; it relies on defining the interface of complexes based on the surface geometry complementarity and amino acid pairwise affinities of the 3D structure of the unbound molecules without prior inclusion of any experimental information [16].

In this study, we explored potential targets and therapeutic agents for T2DM based on scRNA-seq data and transcriptome data of islet *β* cells of T2DM samples and normal samples, the analysis of integrated network pharmacology, and molecular dynamic simulation of drug-protein interactions.

## 2. Materials and Methods

### 2.1. The Source of T2DM Samples and Insulin-Related Genes

The GSE182923 dataset with expression profiles of T2DM samples and the GSE137766 dataset with scRNA-seq data were downloaded from the Gene Expression Omnibus (GEO, http://www.ncbi.nlm.nih.gov/geo) database. The GSE182923 dataset contained ten normal samples and eight T2DM samples. The GSE137766 dataset contained four islet *β* cell samples, where two of them had T2DM features (named as T2DM samples in the following) and the other two of them were nondiabetic samples (named as normal samples in the following). DM-related genes were obtained from DisGeNET (http://www.disgenet.org/) and Comparative Toxicogenomics Database (http://ctdbase.org/). The intersected DM-related genes from the two databases were used as a gene list of DM-related genes in the current study. Insulin-related genes were obtained from the ChEMBL (http://www.ebi.ac.uk/chembl/) database.

### 2.2. Quality Control of scRNA-seq Data

Seurat R package was used to exclude low-quality cells [17]. First, the percentage of mitochondria and rRNA was calculated using the PercentageFeatureSet function, and high-quality cells were screened according to the following quality control criteria: for each cell, the total gene counts should be between 100 and 7500, the mitochondrial gene content was less than 35%, and the number of unique molecular identifiers (UMIs) was more than 1000 (Supplemental Figure S1A-C).

### 2.3. Dimensionality Reduction and Cell Annotation for scRNA-seq Data

The scRNA-seq data was normalized by the log-normalization method in Seurat R package. Then, the FindVariableFeatures function was performed to filter highly variable genes through the analysis of variance (Supplemental Figure S1D). The data was scaled to mean 0 and variance 1, and principal component analysis (PCA) was used for dimension reduction and data visualization. Then, the cells were clustered to different subgroups (clusters) by FindNeighbors and FindClusters functions under conditions of dim = 30 and resolution = 0.1. *T*-distributed stochastic neighbor embedding (*t*-SNE) was utilized to visualize the clustered cells. For screening the marker genes of cell clusters, the FindAllMarkers function was performed to identify the top 5 marker genes of each cluster with log2(fold change (FC)) = 0.5, Minpct = 0.35, and *P* < 0.05.

### 2.4. Enrichment Analysis of Functional Pathway

ClusterProfiler R package [18] was utilized to annotate Kyoto Encyclopedia of Genes and Genomes (KEGG) pathways. ClusterProfiler allows automation of the process of biological-term classification and the enrichment analysis of gene clusters, which supports a universal interface for gene functional annotation from a variety of sources and therefore can be applied in diverse scenarios.

### 2.5. Screening of Important Cell Clusters and Key Genes

The difference between the nondiabetic control group and the T2DM group was analyzed using the Fisher test with the parameter of FC > 4 or FC < 0.25 and *P* < 0.05. The intersection of DM-related and insulin activity-related genes was shown by the Venn diagram. The STRING (https://string-db.org/) database was introduced to conduct protein-protein interaction (PPI) analysis within genes under a confidence score > 0.4. The visualization of the PPI network among the genes was outputted by using Cytoscape software [19]. The genes in PPI with few nodes were removed.

### 2.6. Identification of Hub Gene in Subnetwork

The molecular complex detection (MCODE) algorithm, which can detect closely connected proteins or dense regions of the protein interaction network, was employed to screen important subnetworks contributing to insulin activity and the development of T2DM. KEGG enrichment analysis was performed to identify key subnetworks [20]. Next, Pearson correlation analysis was used to evaluate the relation between the genes within key subnetworks, and immune cell infiltration was calculated by CIBERSORT [21], and hub genes significantly associated with immune infiltration were identified. The receiver operating characteristic (ROC) curve analysis was implemented using pROC package to estimate the performance of the hub genes [22]. The Wilcoxon test was conducted to test the expression of the hub genes between T2DM samples and normal samples. The hub gene with a significant difference between the two groups was considered the final hub gene.

### 2.7. Prediction of CCR5-Related Gene Set and Potential Targeted Drugs

To screen CCR5-related genes in the GSE182923 dataset, rcorr function of Hmisc package (https://cran.r-project.org/web/packages/Hmisc/index.html) was used. The genes with a correlation coefficient > 0.95 and *P* < 0.001 were considered potential target genes for the treatment of T2DM. Based on the drug target pairs obtained from DrugBank (https://go.drugbank.com/) and the key PPI network of STRING (threshold score was 400), the proximity between drugs and T2DM was calculated according to the formula in the published study as follows [23]:

(1) $$\begin{array}{c} d\left(S,T\right)=\frac{1}{\left|T\right|}\underset{t\in T}{\sum }\min_{s\in S}\left(d\left(s,t\right)+\omega \right). \end{array}$$

*S* represents a gene set related to T2DM. *T* is a gene set of drug targets. *d*(*s*, *t*) is the shortest distance between *s* nodes and *t* nodes, where *s* is a T2DM-related gene and *t* is a drug target gene. *ω* indicates the weight of a target gene. *ω* = −ln (*D* + 1) when the target drug gene is within the T2DM-related gene set; otherwise, *ω* = 0.

The significance of relatedness between a drug and hub gene was evaluated using a reference distance distribution corresponding to the drug. Specifically, a group of proteins (*R*) matching the number of drug targets were randomly selected from the network, and the distance *d* (*S*, *R*) between these proteins and genes related to hub genes was calculated. The reference distribution was obtained by repeating the randomization 1000 times. The mean *μ*_*d*(*S*, *R*)_ and standard deviation *σ*_*d*(*S*, *R*)_ of the reference distribution were used to calculate the *z*-score by converting the observed distance to a normalized distance, i.e., proximity value:

(2) $$\begin{array}{c} z\left(S,T\right)=\frac{d\left(S,T\right)-\mu_{d\left(S,R\right)}}{\sigma_{d\left(S,R\right)}}. \end{array}$$

### 2.8. Molecular Docking

CCR5 (PDB ID: 4MBS) was identified as a target to perform molecular docking using AutoDock Vina software using the semiflexible docking algorithm [24]. AutoDockTools 1.5.6 was used to remove excessive protein chains, water, and solvent molecules and add polar hydrogen and charge, etc. The coordinates of the grid box in each direction of *XYZ* during molecular docking were 150 Å, 108 Å, and 22.5 Å, respectively. The length in each direction of *XYZ* was 20 Å. The Lamarckian algorithm [25] was used to recognize the optimal binding mode of ligand molecules with the standards of exhaustiveness = 8, the output conformation number ≤ 10, and the energy range ≤ 3 kcal/mol [26, 27]. The outputted plot was processed by Pymol.

### 2.9. Molecular Dynamic Simulations

The binding stability of the receptor-ligand complex was evaluated by 100 ns molecular dynamic simulations through Gromacs2019 software package [28]. The CHARMm36 force field was used, and the str file of ligands was obtained from the CGenFF program. The system was built in a dodecahedral box, with 0.154 M sodium and chloride ions [29] being added to neutralize the charge. The steepest descent algorithm (no more than 5000 steps) was used to minimize the energy of the solvation system. The LINCS algorithm was applied to constrain covalent bond length, and the electrostatic interaction was calculated by the PME algorithm. Then, 100 ps NVT and NPT simulations were carried out under constant temperature (300 K) and pressure (1 bar) to balance atoms of compounds in an initial coordinate. Finally, 100 ns molecular dynamic simulations were run through Pruduct MD with a time step of 2 fs. The root mean square deviation (RMSD) of the ligands was computed at different times (ns), and the outputted plot was processed by Pymol [30].

### 2.10. Statistical Analysis

R software (v4.0.1) was used to perform all the statistical analyses and R packages. The parameters were defaulted if not specifically indicated. The Wilcoxon test was conducted to test the difference between the two groups. *P* < 0.05 was considered significant.

## 3. Results

### 3.1. Cell Clusters Were Identified in the T2DM scRNA-seq Data

**Figure 1:**
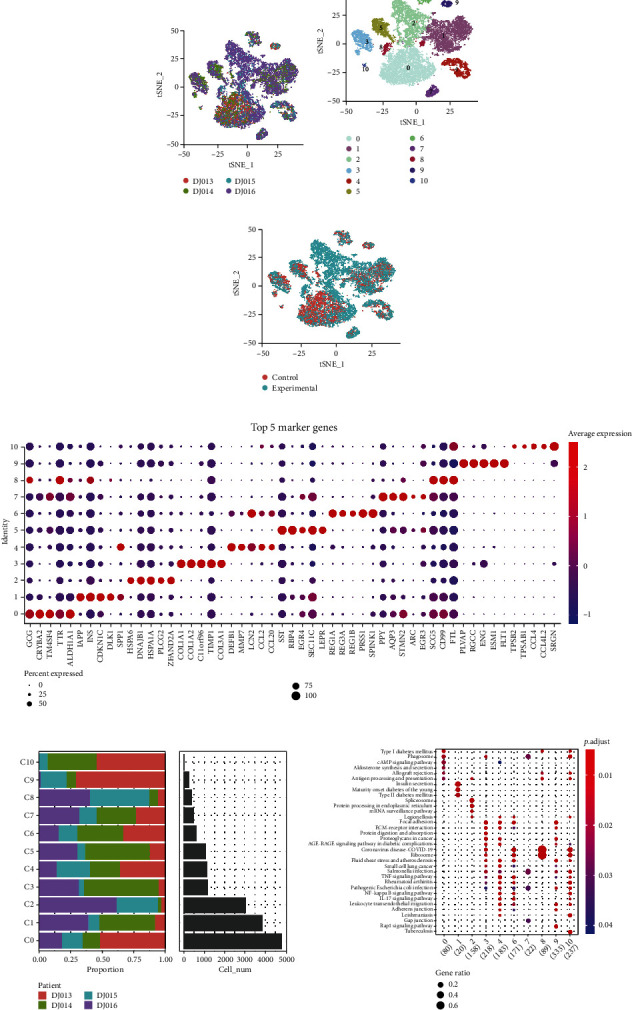
scRNA-seq identified 11 cell clusters in T2DM samples. (a) The *t*-SNE diagram of the cells in each islet *β* cell sample. (b) 11 subgroups of scRNA-seq cluster analysis. (c) The distribution of cells from T2DM samples and normal controls in the subpopulation. (d) The bubble plots show the top five marker genes of each subpopulation. (e) The proportion and cell number of 11 subpopulations in 4 samples. (f) The KEGG analysis of cell enrichment pathway was annotated according to the marker genes of each cluster.

Through preprocessing scRNA-seq data of four islet *β* cell samples in the GSE137766 dataset, 1205 cells and 17106 cells were finally included for further analysis (Supplemental Figure S1, see Sections 2.2 and 2.3). Next, the *t*-SNE algorithm clustered the cells in four islet *β* cell samples into 11 independent subgroups or cell clusters (Figures 1(a)–1(c)). The marker genes of the top five subpopulations were visualized by the bubble chart (Figure 1(d)). C0, C1, and C2 consisted the majority of cell populations, where T2DM samples were significantly accumulated in C2 (Figure 1(e)). In addition, C0, C3, C4, C5, C6, C9, and C10 accounted for a large proportion of normal samples (Figure 1(e)). KEGG enrichment analysis on the marker genes of each cluster revealed that DM-related pathways such as T1DM, T2DM, and insulin secretion were noticeably enriched, where T1DM was enriched in C0, C8, and C10 and T2DM and insulin secretion were both enriched in C1 (Figure 1(f)).

### 3.2. Identification of Key Cell Clusters and Important Marker Genes

**Figure 2:**
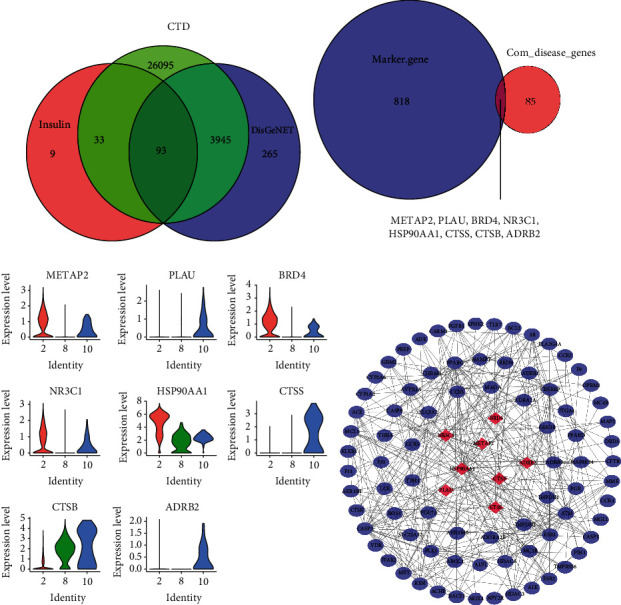
Analysis of important genes related to diabetes mellitus and insulin activity. (a) The Venn diagram shows the intersection of diabetes-related genes and ChEMBL-derived insulin activity-related genes in DisGeNET and Comparative Toxicogenomics Database. (b) Venn diagram displaying the intersection of C2, C8, and C10 marker genes with 93 genes. (c) The expression level of METAP2, PLAU, BRD4, NR3C1, HSP90AA1, CTSS, CTSB, and ADRB2 in C2, C8, and C10. (d) PPI network based on 88 genes. Red rhombus indicates the eight intersected genes associated with both T2DM and insulin activity.

Analysis of differences in 11 subsets of differences of islet *β* cell samples between T2DM patients and normal controls showed that there were three subsets within our threshold range, namely, C2, C8, and C10 (Table 1). Among the 93 genes, 8 belonged to the marker genes of the three key cell clusters, namely, METAP2, PLAU, BRD4, NR3C1, HSP90AA1, CTSS, CTSB, and ADRB2 (Figures 2(a) and 2(b)). The expression of each gene in C2, C8, and C10 was analyzed. The results showed that METAP2, BRD4, and NR3C1 were high-expressed in C2 and C10, that PLAU, CTSS, and ADRB2 were high-expressed in C10, and that HSP90AA1 and CTSB were high-expressed in C2, C8, and C10 (Figure 2(c)). After removing the genes with few nodes in PPI, 88 genes remained and were selected to construct a PPI network, with the above eight genes located at the core of the network (Figure 2(d)).

### 3.3. Regulatory Relationship of Six Key Subnetworks and Their Effects on Infiltrating Immune Cells

**Figure 3:**
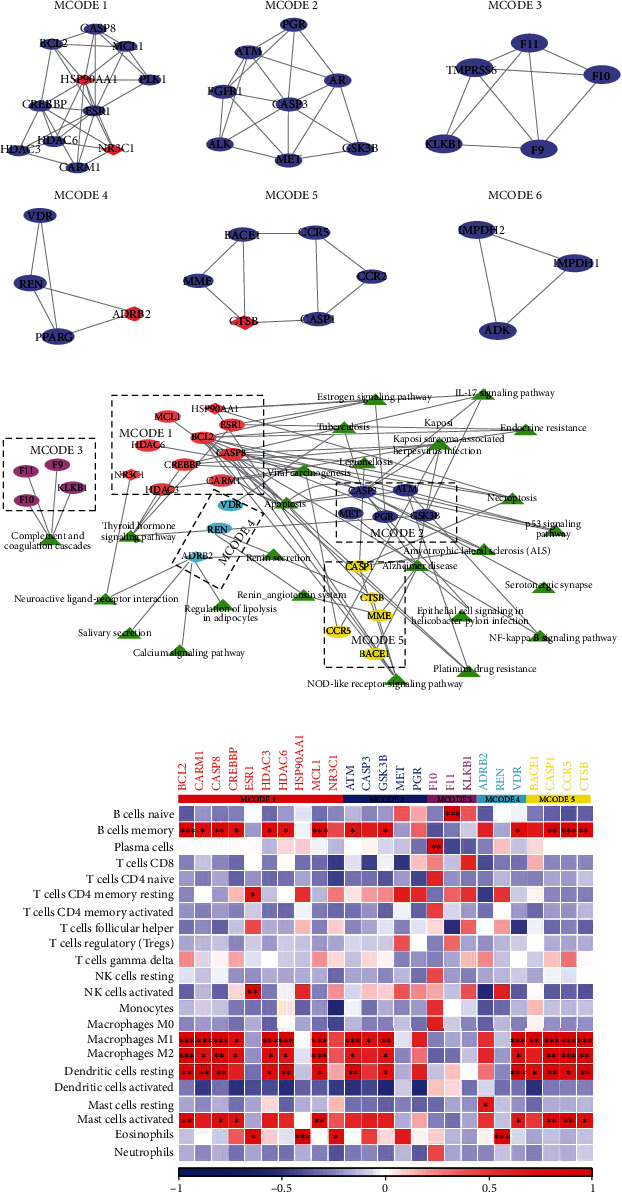
Regulatory relationship of 6 key clusters and their effects on infiltrating immune cells. (a) The key subnetworks in the PPI network were screened based on the MCODE algorithm. (b) KEGG pathways of five key subnetworks (MCODE 1-5). The triangle green represents the pathway, the circle is the disease-related gene, and the diamond is the marker gene of the differential subgroup, and the genes in the same red dotted line belong to the same MCODE. (c) Pearson correlation analysis between each gene within the five key subnetworks and 22 immune cells. Red indicates positive correlation, and blue indicates negative correlation. ^∗^*P* < 0.05,  ^∗∗^*P* < 0.01, and^∗∗∗^*P* < 0.001.

The MCODE algorithm found out six key subnetworks from the PPI network (Figure 3(a)). KEGG enrichment analysis screened the KEGG pathways significantly related to the 88 previously screened genes, among which the genes in MCODE 1-5 were involved in the regulation of these KEGG pathways. Specifically, MCODE 1 was enriched in many signaling pathways, such as apoptosis, Kaposi sarcoma-associated herpesvirus infection, platinum drug resistance, estrogen signaling pathway, and endocrine resistance. MCODE 2 was related to the IL-17 signaling pathway and Alzheimer disease. All the genes of MCODE 3 were enriched in complement and coagulation cascades. The pathways regulated by MCODE 4 include renin secretion, tuberculosis, regulation of lipolysis in adipocytes, and neuroactive ligand-receptor interaction. The gene in MCODE 5 showed many common regulatory pathways with MCODE 1 and MCODE 4, and it also regulated legionellosis alone (Figure 3(b)). Pearson correlation analysis demonstrated that MCODE 1 and MCODE 5 were significantly correlated with memory B cells, M1 macrophages, M2 macrophages, and resting dendritic cells (Figure 3(c)), where BCL2, CARM1, CASP8, HDAC3, HDAC6, MCL1, CASP1, CCR5, and CTSBT2D had significant correlations with all four cell types.

### 3.4. Identification of Genes with the Most Diagnostic Significance for T2DM

**Figure 4:**
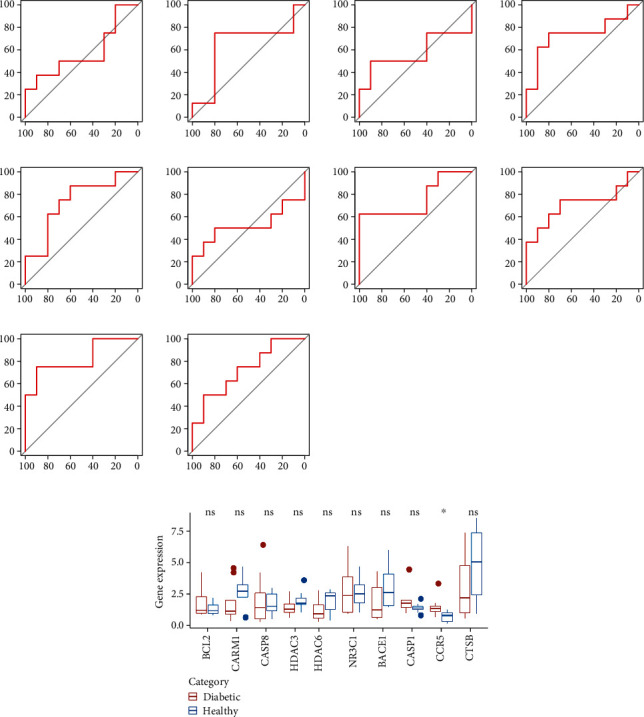
Identification of genes with the most diagnostic significance for T2DM. (a) The ROC curve of diabetes was diagnosed by the expression of BCL2, CARM1, CASP8, HDAC3, HDAC6, NR3C1, BACE1, CASP1, CCR5, and CTSBT2D. (b) The differential expression of 10 genes between healthy samples and T2DM samples was analyzed. The Wilcoxon test was conducted. ns: no significance. ^∗^*P* < 0.05.

Among the genes in MCODE 1-5, the expression of 10 genes (BCL2, CARM1, CASP8, HDAC3, HDAC6, NR3C1, BACE1, CASP1, CCR5, and CTSB) can be detected in the GSE182923 dataset. Evaluating the diagnostic performance of each of the 10 genes showed that the diagnostic efficiency of HDAC3, HDAC6, BACE1, CASP1, CCR5, and CTSB was higher than 70% and that the area under the ROC curve (AUC) of CCR5 was the highest of 82.5% (Figure 4(a)). Among the 10 genes, only CCR5 was differentially expressed between normal samples and T2DM samples (*P* < 0.05, Figure 4(b)). Moreover, we extracted CCR5-related genes, chemicals, and the corresponding pathways (Supplemental Figure S2). The result showed that CASP and CCR protein families closely interacted with CCR5 and that two pathways (viral carcinogenesis and Kaposi sarcoma-associated herpesvirus infection) were enriched.

### 3.5. Identification of Potential Drugs Targeting CCR5

**Figure 5:**
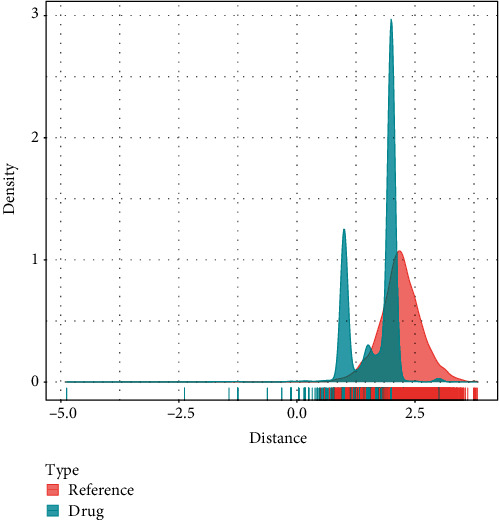
Proximity between drugs and CCR5. Density plot showing the distance distribution of all drugs to PD-related genes (blue) and the reference data (red).

**Figure 6:**
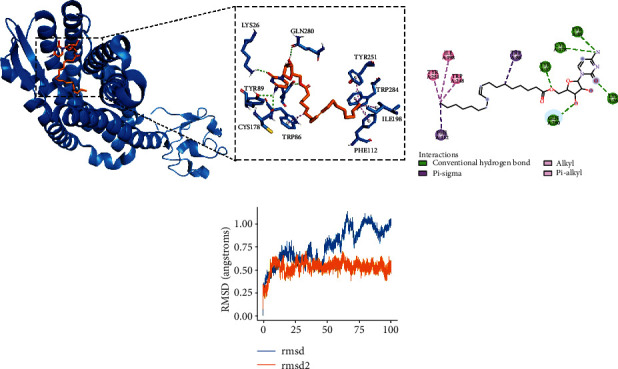
The combination mode of DB05494 and CCR5. (a) The three-dimensional diagram of the interaction between CCR5 and DB05494, the green dotted line represents the hydrogen bond, and the purple dotted line represents the hydrophobic interaction. (b) 2D diagram of the interaction between DB05494 and CCR5. (c) The RMSD of the simple protein (1-blue) and molecular complex (2-orange).

To identify potential drugs targeting CCR5, 49 genes significantly related to CCR5 in GSE182923 were screened. Then, the proximal drugs were collected by the application of web-based proximity analysis, and the drugs without a significant correlation with CCR5 were excluded. By mapping CCR-related genes to the network, the proximity of drugs to CCR5-related genes was calculated. The density map showed that the distance distribution between drugs and CCR5-related genes showed partial overlap but was smaller than that of reference background drugs and CCR-related genes (Figure 5). We selected drugs with global FDR < 0.001 and obtained 145 drug candidates. The first five drugs with the smallest distance were DB05494, DB00629, DB01018, DB02594, and DB00484. The docking score of each drug with CCR was calculated, and we detected that the docking score between DB05494 and CCR5 was determined to be -8.0 kcal/mol (Table 2). DB05494 produces hydrogen bonds with LYS26, TYR37, TYR89, CYS178, and GLN280 of receptor proteins and hydrophobically interacts with TRP86, PHE112, ILE198, TRP248, and TYR251 of CCR5 (Figures 6(a) and 6(b)). In the 100 ns molecular dynamic simulation, DB05494 binding to CCR5 showed light microwave motion from 0 to 10 ns. When compared to the simple protein, the simulation from 10 to 100 ns showed a highly stable ligand RMSD (Figure 6(c)). These results indicated that DB05494 could stably conjugate with CCR5 and exert potential biological activity. Moreover, docking the ligand of 4MBS with CCR5 showed a significantly low root-mean-square distance (RSMD) of 0.4550 Å that was far below 2 Å (Supplemental Figure S3), supporting the reliability of our docking method.

## 4. Discussion

T2DM is a common chronic illness characterized by insulin resistance and eventually by decreased insulin secretion by pancreatic beta cells, leading to chronic hyperglycemia and associated long-term disease complications. The rising burden of T2DM remains one of the biggest health challenges today [31]. So far, T2DM is still incurable, and many drugs do not always fully control T2DM unless they are used or combined used in the early stages of disease progression [32]. However, this strategy may be affected by issues associated with the multipharmacological methods, such as some side effects, toxicity, and unwanted drug-drug interactions. An alternative strategy is to use a single drug to selectively modulate different targets, which may have the potential to improve the balance between efficacy and safety compared to single-target drugs [33].

In this study, we designed a systematic computing framework and used network analysis to identify potential therapeutic drugs and drug targets of T2DM. Firstly, we collected insulin-related genes and T2DM-related genes from two databases and developed a PPI network based on the genes coexisting in the three databases. From the PPI network, we found 6 key MCODEs. The genes in MCODE, individually or jointly, regulated a wide range of biological pathways, either individually or jointly, including apoptosis, platinum drug resistance, estrogen signaling pathway, endocrine resistance, complement and coagulation cascades, renin secretion, and regulation of lipolysis in adipocytes. The relationship between most of these pathways and the development of diabetes have been reported in previous studies. It has been shown that the estrogen signaling pathway regulates rapid changes in systemic metabolism, fat distribution, and insulin action associated with diabetes [34]. Diabetes is caused by metabolic disorders of the endocrine system [35]. Previous studies have demonstrated that androgen receptor-mediated endocrine resistance was associated with diabetes [36]. A direct positive correlation between blood glucose control and renin has been reported. Hyperglycemia increased succinate concentration and succinate receptor activation in the kidney, leading to renin release [37]. Regulation of lipolysis in adipocytes is closely related to insulin resistance. Inhibition of adipocyte lipolysis could be a promising treatment strategy for T2DM related to insulin resistance and obesity prevention [38]. Therefore, the genes in MCODE are likely to be involved in the regulation of these pathways to affect the progression of diabetes.

Among the MCODE genes screened above, we found that BCL2, CARM1, CASP8, HDAC3, HDAC6, MCL1, NR3C1, BACE1, CASP1, CCR5, and CTSBT2D were expressed in another independent dataset (GSE182923) with T2DM samples. Only the expression of CCR5 was significantly different from that in normal samples, with the highest AUC of 82.5%. CCR5 is a kind of chemokine receptor (CCR), and it is known to contribute to metabolic disorders such as T2DM, obesity, atherosclerosis, and human immunodeficiency virus (HIV) infection-induced metabolic changes [39]. Some studies have reported the association of CCR5 polymorphism with clinical outcomes of T1DM, T2DM, and diabetic complications. There was a correlation between CCR5-del32 mutation and the clinical course of T1DM, which was the pathogenic factor of T1DM [40]. [41] reported that the expression of CCR5 in T2DM patients was increased by measuring the peripheral blood mononuclear cells, and CCR5 can be considered an indicator of atherosclerosis in diabetic people by promoting insulin resistance and aggravating pancreatic beta cell dysfunction during the development of diabetes. Muntinghe et al. [42] reported that the existence of CCR5-delta32 was associated with the high survival rate of patients with T2DM. These results are similar with that reported by Slominski et al. who showed that CCR5-delta32 polymorphism is related to celiac disease and autoimmune thyroiditis coincidence in patients with T1DM [43]. Moreover, the variation of CCR5 was associated with the susceptibility to nephropathy in patients with T2DM [44]. Considering the relationship between CCR5 and T2DM, we screened 49 genes associated with CCR5 in the dataset to explore and find multitarget drugs that simultaneously target these genes. By analyzing the network-based proximity between drugs and CCR-related genes, we obtained 145 drug candidates and calculated the binding energy of the first five genes with the smallest distance to CCR5. Finally, the DB05494 with the smallest score of docking with CCR5 was selected for simulated molecular docking. It has been observed that DB05494 produced hydrogen bonds with LYS26, TYR37, TYR89, CYS178, and GLN280 of CCR5 and hydrophobically interacts with TRP86, PHE112, ILE198, TRP248, and TYR251 to form a stable conformation. This indicated that DB05494 may be a potential drug targeting CCR5.

There are limitations to this study. Firstly, T2DM samples in single-cell data are transited from nondiabetic islet *β* cells infected by adenovirus (packaging with T2DM-related transcriptional factors that can result in T2DM-like feature) [45]. The nonoriginal T2DM samples may affect the accuracy of the analysis. Nevertheless, Gribov et al. [17] demonstrated that T2DM-like islet *β* cells could be reversed to nondiabetic islet *β* cells, supporting the reliability of the cell model. Therefore, to some extent, the analysis based on T2DM-like samples was reasonable and reliable. Secondly, although DB05494 is a potential therapeutic candidate for treating T2DM, whether it is effective against T2DM has not been confirmed by in vitro or in vivo experiments. In addition, CCR5 is a well-known target for treating HIV infection [46], and it is also a potential target for neuroinflammatory diseases [47]. Whether targeting CCR5 in T2DM patients may induce other inflammatory responses remains unclear. On the one hand, expanded biological experiments are needed to demonstrate the exact role of CCR5 blockade and the efficiency of DB05494 in inhibiting CCR5 for T2DM treatment. On the other hand, the adverse effects of targeting CCR5 such as on the aspect of immune response should also be verified.

## 5. Conclusion

In conclusion, our work combined scRNA-seq data and transcriptome data of T2DM samples and identified CCR5 as a potential target for T2DM treatment based on PPI network analysis. Through employing molecular docking and simulative molecular dynamics, DB05494 was screened as a potential therapeutic drug for treating T2DM. Our study clarified the potential role of CCR5 in T2DM from comprehensive bioinformatics analysis and supported the previous findings that CCR5 variants were associated with the development of diabetes.

## Figures and Tables

**Table 1:** Differences of 11 subgroups in islet *β* cell samples between T2DM patients and nondiabetic controls.

Cell_name	*P*.val	Adjust.*P*.val	fc
C0	8.20*E* − 140	4.51*E* − 139	0.41759
C1	0.002008	0.002209289	0.892653
C2	0	1.46*E* − 14	22.39432
C3	1.10*E* − 24	0	0.533703
C4	1.40*E* − 11	2.73*E* − 24	0.660653
C5	1.95*E* − 20	1.72*E* − 11	0.554438
C6	1.24*E* − 24	3.06*E* − 20	0.422659
C7	0.061583	2.73*E* − 24	0.842438
C8	1.96*E* − 60	0.061582664	6.80658
C9	2.12*E* − 22	7.20*E* − 60	0.274513
C10	1.06*E* − 14	3.89*E* − 22	0.076347

**Table 2:** Docking results of CCR5 with five compounds.

Compound	Score (kcal/mol)	Hydrogen bond	Hydrophobic bond
DB05494	-8.0	LYS26, TYR37, TYR89, CYS178, GLN280	TRP86, PHE112, ILE198, TRP248, TYR251
DB00629	-6.5	TYR37, GLU283	TYR108, TRP86, TYR89
DB01018	-7.1	TYR37, GLU283	TRP86, TYR89, TYR108
DB02594	-5.8	TYR37	TRP86, TYR108
DB00484	-6.9	—	TRP86, TYR89, TYR108, THR167

## Data Availability

The datasets used and/or analyzed during the current study are available from the corresponding author on reasonable request.

## Supplementary Materials

Supplementary Materials Supplemental Figure S1: preprocessing scRNA-seq data of four T2DM samples. Supplemental Figure S2: CCR5-acted compounds and biological networks. Supplemental Figure S3: molecular docking between the ligand of 4MBS and CCR5. (Supplementary Materials).
