# Supplementary material for: Comprehensive Network Analysis Reveals the Targets and Potential Multitarget Drugs of Type 2 Diabetes Mellitus
Source: Oxid Med Cell Longev. 2022 Jul 28;2022:8255550. doi: 10.1155/2022/8255550 (PMC9352488; doi:10.1155/2022/8255550)
Supplement: Supplementary Materials — Supplemental Figure S1: preprocessing scRNA-seq data of four T2DM samples. Supplemental Figure S2: CCR5-acted compounds and biological networks. Supplemental Figure S3: molecular docking between the ligand of 4MBS and CCR5. (Supplementary Materials). [file 8255550.f1.docx]

**Supplementary Figure S1. Preprocessing scRNA-seq data of four T2DM samples**

A: Correlation analysis, including the correlation between UMI and mRNA, the correlation between UMI and mitochondrial gene content, the correlation between mRNA and mitochondrial gene content. The number on the axis is the value of R. B, C: Low quality cells were eliminated by quality control from T2D islet β cell samples from 2 infected and uninfected adenovirus. D: Preliminary PCA dimensionality reduction of scRNA-seq data.


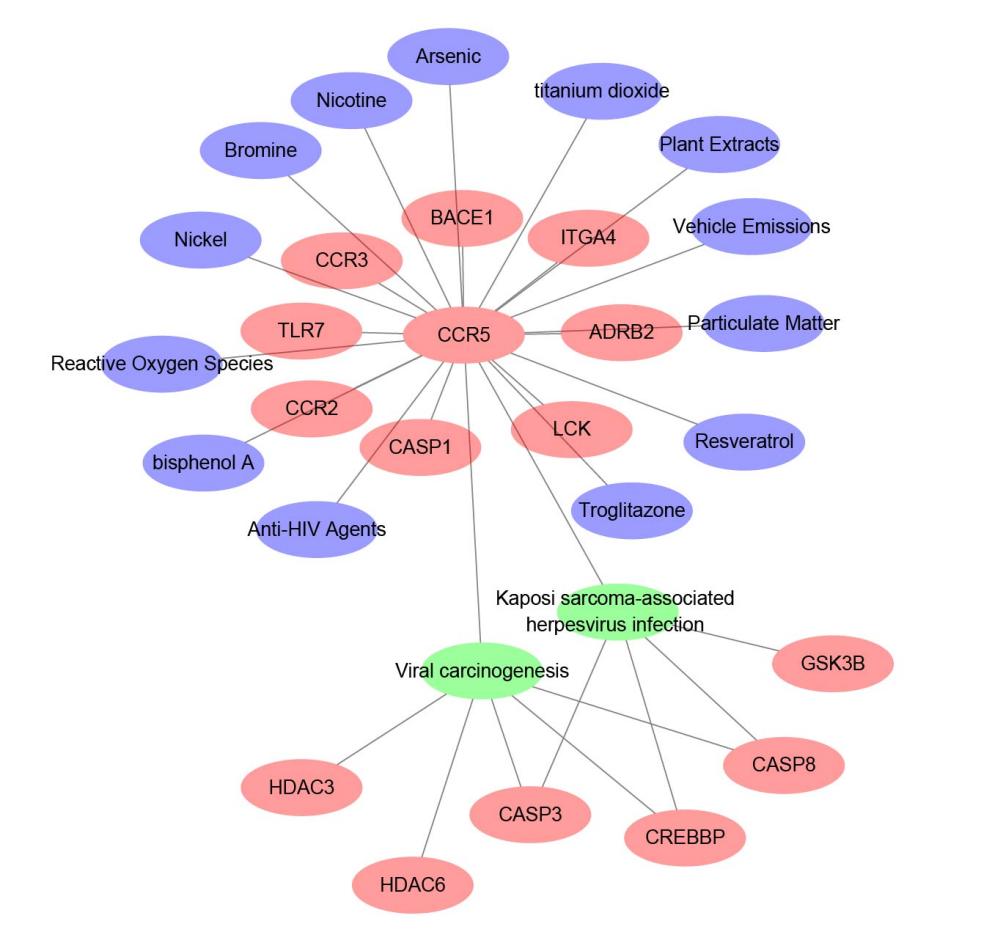


**Supplementary Figure S2. CCR5-acted compounds and biological networks.** Red ellipses represent genes, blue ellipses represent inference chemical name, and green ellipses represent signal pathways.


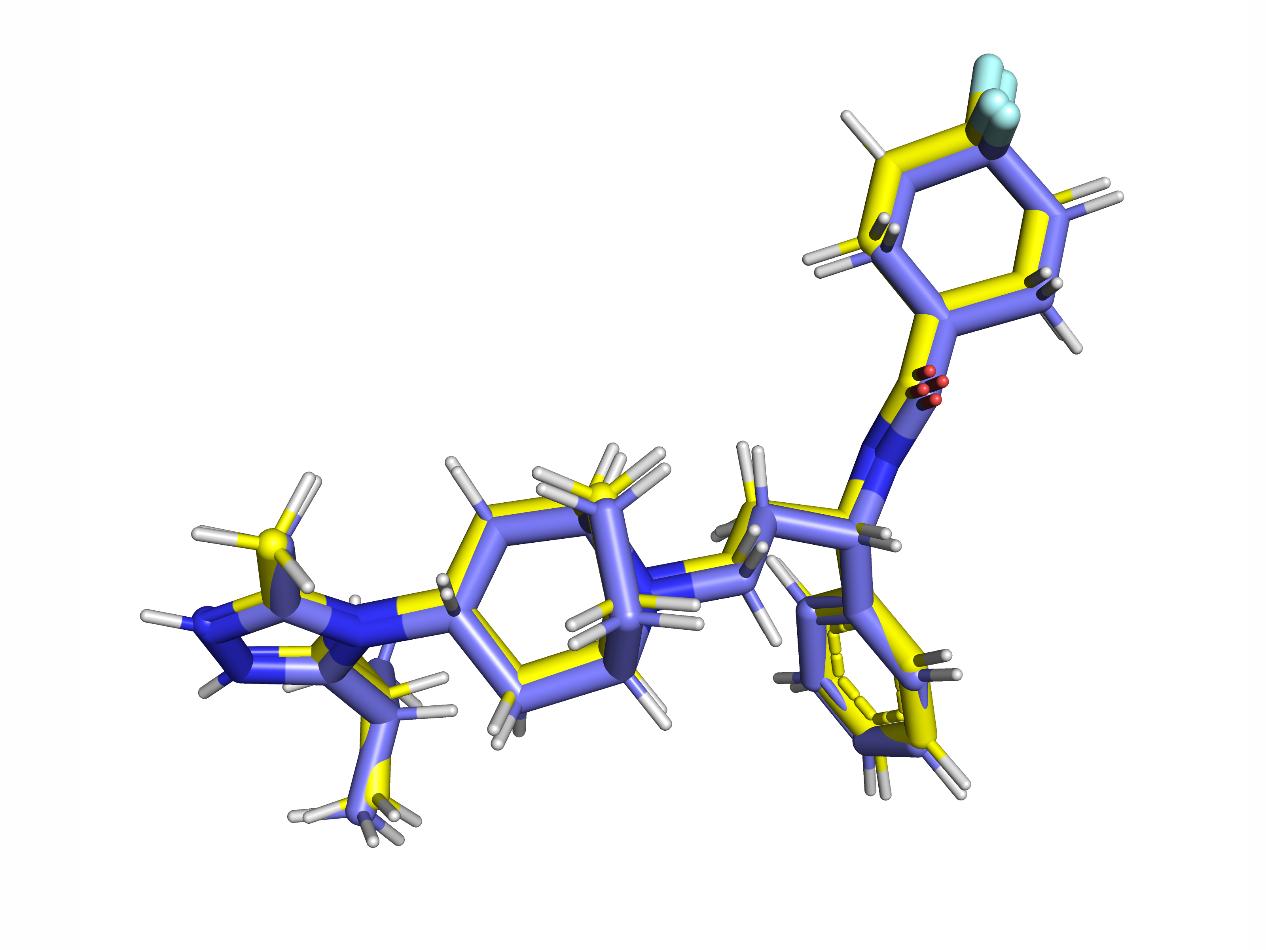


**Supplementary Figure S3. Molecular docking between the ligand of 4MBS and CCR5.**
